# SPOUSAL MULTIMORBIDITY AND DEPRESSIVE SYMPTOMS AMONG OLDER INDIAN COUPLES: DOES ONE’S OWN HEALTH AND GENDER MATTER?

**DOI:** 10.1093/geroni/igad104.2429

**Published:** 2023-12-21

**Authors:** Soohyeon Ko, Rockli Kim

**Affiliations:** Korea University, Seoul, Republic of Korea; Korea University, Seoul, Republic of Korea

## Abstract

With the aging population, increases in non-communicable diseases that require chronic management pose economic and social burdens to individuals with multimorbid conditions and their spousal caregivers. However, little is known about the crossover effect of spousal multimorbidity on mental health outcomes in the context of low-and middle-income countries, and whether it depends on one’s own health status and gender. We used data on 6,158 older couples (12,316 individuals aged 60 years or above) from the Longitudinal Aging Study in India (LASI) 2017-18 to examine the association between spousal multimorbidity and depressive symptoms. Overall, 23.4% of the sample were multimorbid and 27.0% reported having depressive symptoms in the past week. Multivariate logistic regression models showed that spousal multimorbidity was associated with depressive symptoms, even after accounting for one’s own multimorbidity status (adjusted odds ratio(aOR)=1.23; 95% confidence interval (CI)=1.06-1.44). However, this association was gender-specific. Among men, when jointly considered, spousal multimorbidity was no longer statistically significant whereas their own multimorbidity was associated with 60% higher odds of having depressive symptoms (aOR=1.60; 95% CI=1.28-2.00). Furthermore, the association between spousal multimorbidity and depressive symptoms was contingent upon the presence of their own multimorbidity for men. Among women, spousal multimorbidity was significantly associated with depressive symptoms, regardless of their own multimorbidity status. Our findings indicate that interventions to promote healthy aging should expand the formal caregiving system and consider family-based approaches to minimize the crossover health consequences of morbidity in conjugal relationships, especially for women.

